# A secularly varying hemispheric climate-signal propagation previously detected in instrumental and proxy data not detected in CMIP3 data base

**DOI:** 10.1186/2193-1801-1-68

**Published:** 2012-12-15

**Authors:** Marcia Glaze Wyatt, John M Peters

**Affiliations:** 1 Department of Geologic Sciences, CIRES/INSTAAR, University of Colorado-Boulder, Benson Earth Sciences Building, Boulder, CO 80309 USA; 2 Department of Mathematical Sciences, Atmospheric Sciences Group, University of Wisconsin-Milwaukee, Milwaukee, WI 53201-0413, USA

**Keywords:** Climate, Network, Synchronize, CMIP3, Stadium-wave, Signal propagation

## Abstract

Results of previous studies support the existence of a spatially coherent, secularly varying climate signal, propagating through a network of synchronized climate indices across the Northern Hemisphere during the 20^th^ century. The signal was identified in both instrumental and proxy data sets. In this present study, we seek to detect this same low-frequency signal propagating hemispherically through networks of model-simulated climate indices. These simulated climate indices were reconstructed from a data set generated by models of the third Coupled Model Intercomparison Project (CMIP3). Methods used in the earlier studies on climate-signal propagation guide the strategy for this companion study, for which 60 network analyses were performed. Most analyses focused on 20^th^ century behavior, several on pre-industrial conditions. None succeeded in reproducing a hemispherically propagating signal. In light of previous results, we offer possible reasons for this finding. Among them is speculation on whether mechanisms relevant to signal propagation might be missing from this suite of general circulation models.

## 1 Introduction

Recent work using instrumental data of the 20^th^ century suggests that a spatially coherent, low-frequency climate signal propagates across the Northern Hemisphere (Wyatt et al. 2011). Authors of this 2011 paper analyzed a lagged covariance structure of a network of eight climate indices. Their results detailed the transmission of a multidecadal-scale climate signal propagating throughout the Northern Hemisphere through a sequence of synchronized^a^ atmospheric and lagged oceanic teleconnections. The authors termed this signal propagation the ‘stadium wave’ - a term alluding to the behavior often seen in a sports arena, where successive groups of spectators stand with arms raised, and then sit, giving the visual impression of a wave passing through the crowd.

Subsequent dissertation work by Wyatt ((2012) and submitted manuscript (2013)) probes both spatially expanded data sets of geophysical indices and temporally expanded data sets of proxies (1700 to 2000). Hemispheric signal propagation is found in all sets. All network combinations of twentieth-century data, both proxy and instrumental, reflect consistent results of apparent quasi-periodicity and signal propagation. Prior to 1850, signal propagation is evident; yet time scale of variability in the proxy-index networks differs slightly from the time scale of variability exhibited by the signal seen in all data sets post-1850.

This latter observation brings up an important point. The significant finding regarding the ‘stadium-wave’ signal identified in these diverse index sets is its propagating signature. Timescale of its variability can be characterized as low-frequency, but we stop short of claiming periodicity, or even quasi-periodicity. This we cannot statistically assess. While all 20^th^-century data sets - the originally used eight-member instrumental set, the 20^th^ century portion of the proxy set, and the spatially expanded instrumental set - reflect similar secular-scale^b^ variability centered at ~64 years over this century-scale interval; a one-hundred-year time series is too short a quantity over which to claim identification of a statistically significant multi-decadal-scale periodicity. In addition, proxy data sets, while longer, are inherently noisy, thereby adding challenge to statistical significance assessment. These points made, it is hard to ignore the pervasiveness of multidecadal (~50 to 70-year) fluctuations identified in records of diverse, and perhaps indirectly related indices: from numerous and varied climate-related parameters (e.g. Kushnir 1994; Minobe 1997, Minobe 1999; Klyashtorin and Lyubushin 2007; Frolov et al. 2009 and references therein; Nowak et al. 2011; Chambers Don et al. 2012) to similarly cadenced variations in commercial-fish populations (Beamish and Bouillon 1993; Beamish et al. 1997, 1999; Chavez et al. 2003; Klyashtorin 1998; Klyashtorin and Lyubushin 2007; Klyashtorin et al. 2009), cosmogenic nuclide accumulations (Ogurtsov et al. 2002; Patterson et al. 2004), geomagnetic-field intensity (Courtillot et al. 2007, Roberts et al. 2007), Earth’s rotational-rate anomalies (Beamish et al. 1999; Sidorenkov et al. 2005; Sidorenkov 2005, Sidorenkov 2009), and solar-related aurora records of the mid-latitudes (Scafetta 2011).

While the authors of the previous stadium-wave studies were unable to assign statistical significance to a quasi-oscillatory nature of the identified signal in observed and proxy data, they were able to quantify the likelihood that a low-frequency signal, characterized by delayed alignment of spatially and dynamically diverse indices, i.e. a hemispherically propagating signal, could be due to mere random chance. That likelihood was found to be less than 5% in observational data sets for the 20^th^ century.

Beyond instrumental and proxy data, the last realm of data available to us is model-generated data. We want to know if further support for the stadium-wave signal can be identified by applying our statistical methods to simulated indices reconstructed from model-generated raw variables.

Section 2 details the methods and data; section 3 provides the results; section 4 offers discussion of results; and 5 presents the conclusion.

## 2 Approach, data sets, and methods

### 2.1 Approach

The strategy underlying all stadium-wave studies involves evaluating collective behavior within a network of synchronized interacting nodes. In the case of climate, these nodes represent climate indices - regional patterns of ocean, atmosphere, or ice dynamics, for examples. Viewing systems as networks is common in many disciplines, from biology to electronics to social sciences. Fundamental to networks is the observation that behavior of a system does not equal merely a sum of component parts. The difference between the collective behavior of interacting parts versus a collection of behaviors of individual parts can be traced to how those parts (nodes) are linked (coupled). The latter determines communication. Communication is at the core of a network’s breadth and stability (e.g. see Pikovsky et al. 2003).

This present model-based inquiry is an extension of previous instrumental and proxy work on the ‘stadium-wave’ climate signal. We repeat here the methodology followed in those studies and use those results to guide our interpretation of results derived from the model data.

### 2.2 Data sets

Five steps were involved in data preparation. Details follow.

#### 2.2.1 Acquiring the raw model-simulated data

Data sets of model-generated raw variables - e.g. sea-surface temperature (SST), sea-level pressure (SLP), sea-surface height (SSH), wind strength and direction, etc. - were obtained from the third Coupled Model Intercomparison Project (CMIP3: Meehl et al. 2007) web site (https://esg.llnl.gov:8443/about/registration.do) - a site comprising a vast collection of data sets generated by atmospheric-oceanic general circulation models used in Intergovernmental Panel on Climate Change (IPCC)-related projects. Acquisition of data from this site is available to all researchers, requiring only registration for its use.

Twenty-two models are represented by CMIP3. Dozens of experiments have been performed by each model, most with several runs each. Two experiments were of interest to us: 1) 20^th^-century runs and 2) control runs. Twentieth-century CMIP-model runs generally cover the historical period 1850 to 2000. Such model runs incorporate observed radiative forcings (greenhouse gases (with CO2 increases of 1% per year), aerosols, volcanic eruptions, etc.) throughout the period, the exact levels and proportions of forcings differing among models (Reichler and Kim 2007). In contrast, control experiments, also termed “long controls”, generally cover 500-plus years. Incorporated in the long controls are atmospheric forcings that are held constant, in particular, that of CO2, which is held at pre-industrial levels of 280 parts per million (ppm).

We considered all 22 models participating in the CMIP data base. For most model runs, data are available on daily, monthly, and annual bases. We were interested in monthly records. Data were extracted via varied codes custom-tailored to the format and size of data files. Once downloaded, these data were compiled in variable-specific files for subsequent use. Upon completion of this data-acquisition step, we had successfully extracted data from 21 of 22 models. Information from one model was unavailable. We evaluated at least one 20^th^-century experiment for each of the 21 models. For several of these, analysis of a second run of the 20^th^-century experiment was performed. In addition, for six of the models, we analyzed one run of a pre-industrial control experiment.

#### 2.2.2 Reconstruction of indices

For the stadium-wave studies, in order to evaluate collective behavior, we compact raw-variable data into nodes, or climate indices. Sacrifice of some phenomenological detail due to this data compression is compensated for by advantages that include potentially increased interpretability, enhanced statistical significance, and an apparent increase in signal-to-noise ratio.

Selection of indices for this study parallels the index selection in the original study. This selection includes: the Northern Hemisphere averaged-surface temperature (NHT), the Atlantic Multidecadal Oscillation (AMO), the North Atlantic Oscillation (NAO), a sea-surface-temperature-anomaly-based index of the El Nino Southern Oscillation (NINO3.4), the Pacific Decadal Oscillation (PDO), the North Pacific Oscillation (NPO), and the Aleutian Low-Pressure Index (ALPI). An eighth index was used in the original instrumental-based study: atmospheric-mass-transfer anomalies (AT). AT is an index representing dominant flow direction of large-scale wind fields over the mid-to-high latitudes of the North Atlantic basin and the Eurasian continent. It reflects longitudinal shifts in position of atmospheric centers-of-action and is considered a proxy for atmospheric-heat transfer. Reconstruction of this index proved impractical with the CMIP model-generated data; thus it was omitted from this study. We cannot claim to know if results of our work would have been the same with this index’s inclusion, but we find rationale in its exclusion based on results of a previous study by Wyatt (2012).

In that previous study, Wyatt analyzed an expanded collection of instrumental and proxy data. The set consisted of the original eight stadium-wave indices plus about a dozen additional indices representing diverse geographical regions and geophysical processes. Wyatt performed analyses on the full set, as well as on numerous subsets, some of which did not include AT. A statistically significant stadium-wave signal was identified in each assembled data set for the 20^th^ century. The index, AT, was not necessary for signal expression; although statistical significance was increased with its inclusion.

To construct our model-based network of seven indices^c^ out of monthly values of model-simulated raw variables, we use index-specific codes. For example, to reconstruct a monthly sampled time series for the AMO index, we rely on its standard definition (e.g. (Sutton and Hodson 2003) (see Table 1)). Following this conventional AMO construction, for each month of each year considered we averaged the model-generated SST anomalies over the region from 0° to 60°N and from 75°W to 7.5°W. This and all index codes used in this study are readily available in the literature and in Table 1 in this paper.

**Table 1:** **Indices used in study and descriptions for index-reconstructions**

Index	General description for index-reconstruction:	Reference
NHT	Average surface land temperature and SST of the Northern Hemisphere	(Jones & Moberg 2003; Rayner et al. 2006)
AMO	SSTA averaged over 0 to 60°N, 75°W to 7.5°W	(Kerr 2000; Enfield et al. 2001; Sutton and Hodson 2003)
NAO	Normalized pressure (SLP) difference between the Azores High-Pressure system (~33.2°W, 32.2°N) and the Icelandic Low-Pressure system (~33.15°W, 59.6°N). [~1^st^ PC of SLP in North Atlantic]	(Hurrell 1995)
NINO3.4	Average SSTA 5°N to 5°S, 170°W to 120°W	(Trenberth 1997)
NPO	2^nd^ EOF of SLPA from 100°E to 120°W, 0 to 90°N	(Wang et al. 2007)
PDO	Leading PC of SSTA north of 20°N in North Pacific; globally averaged SSTA subtracted from SSTA in Pacific to remove global-warming signal.	(Mantua et al. 1997)
ALPI	Relative intensity of SLP in North Pacific around 50°N in winter (DJFM). The mean area in km^2 with SLP less than 100.5kPa. Expressed as anomaly relative to 1950 to 1997 mean.	(Beamish et al. 1997)

#### 2.2.3 Computing boreal-winter index averages

From the monthly values of reconstructed indices (section 2.2.2), boreal-winter month (December, January, February, March (DJFM)) values were extracted and averaged. We chose this seasonal interval, as was done in previous stadium-wave studies (where possible). This is a time when atmospheric circulation is most intense and atmospheric modes most pronounced. The boreal-winter mean values for each year were used to construct annually based time series for all reconstructed indices.

#### 2.2.4 Choice of index-time-series length

The majority of analyses (section 2.3) in this study were applied to 20^th^-century model-simulated climate indices. For these analyses, we duplicated the original stadium-wave study’s methodology exactly. This required that all time series for this collection of models were truncated; only the years 1900 to 1999 were used. In the cases where pre-industrial (control or long runs) experiments were analyzed, timescales were longer. For these special cases, we varied time-series lengths of reconstructed indices. Lengths varied from 150 years to 500 years.

#### 2.2.5 Linear de-trending and normalization to unit variance of index time series

Once a model run’s seven-member annually represented (boreal-winter-mean) index set was complete, and prior to analysis, time series of each index were linearly detrended (least-squares method) and divided by the index’s standard deviation to normalize it to a unit variance.

Annual (boreal-winter mean) samplings were used on all iterations. On series over 100 years, both annual sampling and five-year smoothing were applied. A total of 60 analyses were completed on these time series. Methodological details of these analyses follow.

### 2.3 Methods

#### 2.3.1 General description of multivariate statistical method

To these simulated networks of prepared reconstructed-index time series, we applied the multivariate statistical method, Multi-channel Singular Spectrum Analysis (M-SSA: (Broomhead and King 1986; Elsner and Tsonis 1996; Ghil et al. 2002)), to extract and characterize dominant spatio-temporal patterns of data shared by our networks of indices. A generalization of Empirical Orthogonal Function (EOF; Preisendorfer 1988) analysis, M-SSA excels in its ability to detect a signal propagating through a collection of indices (Ghil et al. 2002 and references therein) and is particularly effective in picking up a signal from relatively short, noisy data sets. The reader is directed to http://en.wikipedia.org/wiki/Singular_Spectrum_Analysis where a good description of this relatively standard and increasingly utilized method is given, relevant formulas outlined, and further references offered.

In brief, M-SSA is used to decompose a multivariate time series into oscillatory components and noise. Within this noise, a low-frequency signal is possible. In the case of high-frequency periodic oscillations and low-frequency secularly varying trends, for either to be considered as signals distinct from noise, significance testing, tailored to the signal’s time scale, must show the null hypothesis of random occurrence likely to be rejected.

#### 2.3.2 Preparing the index data set for M-SSA application

Prior to M-SSA application, an extended matrix is constructed for each model-simulated index network. Columns of index time series are referred to as channels. The time series of each of the seven indices is augmented by M shifted, or lagged, copies thereof. M=20 is used in this study^d^. Lag-window size, as represented by M, is chosen based mostly on time series length and partly on the range of periodicities of variability one expects to find in the data. This will be re-visited in section 2.3.3.

#### 2.3.3 Identifying patterns of simultaneous and lagged co-variability in an index network

**Figure 1:**
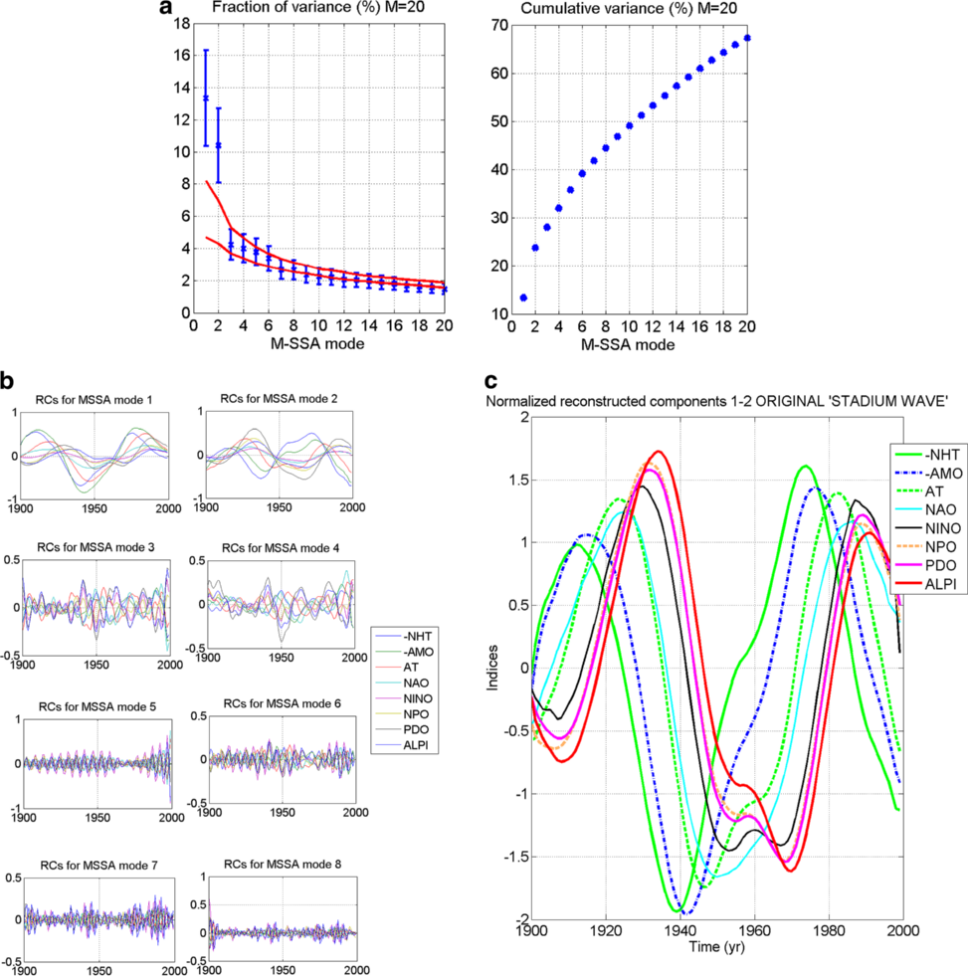
**Statistical Results from Original Stadium-Wave analysis, given here for comparison to model results.** (**a**) Shows individual variances (%) and (b) cumulative variances (% of the total) of the modes of variability shared by the collection of eight instrumental climate indices. The M-SSA window size is M=20. The error bars in (**a**) are based on (North et al. 1982) criterion, with the number of degrees-of-freedom set to 40, based on the decorrelation time scale of ~2.5 years. The red lines in panel (**a**) represent the 95% spread of M-SSA eigenvalues based on 100 simulations of the eight-valued red-noise model (1), which assumes zero true correlations between the members of the eight-index set. Note the leading two modes of variability; they are widely separated from the remaining modes; their error bars overlap. (**c**) Depicts reconstructed components (RCs) for each of the eight modes of variability derived. RCs of the leading modes show similar variability. (**d**) Normalized reconstructed components (RCs) of M-SSA leading two modes of variability are plotted. Note that each index carries this signal, and that the signal propagates through the network of regionally diverse indices. (Adapted from Wyatt et al. 2011). RC time series have been normalized to have unit variance. Note: RCs of NHT and AMO are negative.

M-SSA is applied to each network’s extended data matrix. Patterns, or modes, of variability shared by all network members at a zero or non-zero lag are thereby identified. In the stadium-wave studies, each index time series represents a spatial region. Because of the network’s spatial character, an eigenfunction – a function that best describes the shared pattern of variability – of this extended lagged covariance matrix provides a spatio-temporal filter. It is through these filters that patterns of hemispherically shared climate variability are defined within our index network. The mode whose pattern explains the most variance in the extended time series is the leading mode – mode one. The variances explained by modes two, three, etc. decrease progressively as the mode numbers increase. Mean values of leading modes are then plotted on an M-SSA spectrum. Figure 1a shows an example of an M-SSA spectrum adapted from (Wyatt et al. 2011). This spectrum identifies the first ten leading modes of variability identified in the original stadium-wave data set. Note that modes one and two stand out far from the remaining modes. Figure 1b depicts the cumulative variance accounted for by the first twenty modes identified in that original study.

If one is seeking oscillatory patterns, one looks to the plotted M-SSA spectrum. A pair of leading modes, both well separated from all remaining modes, and whose variances and periodicities are similar to one another, presents potential. But a caveat arises. Only those identified pairs with a periodicity less than or equal to M (usually set at about one-fifth of the time-series length) can be tested for statistical significance (Allen and Robertson 1996) of the detected oscillation period.

But this limitation does not preclude extraction of low-frequency behavior. In addition to the M-SSA-extracted higher frequency oscillations, low-frequency patterns of network co-variability, both simultaneous and lagged, can be mined from the residual data. Such patterns can be expressed through a leading single mode or through a combination of leading modes of similar variance and frequency. And while those patterns exhibiting time scales of variability greater than the value of M cannot be tested for statistical significance in the context of an oscillatory pair (Allen and Robertson 1996); they can be considered to be secular-scale trends. Often, such secularly varying trends can be attributable to red-noise, i.e. randomness. But randomness is not always the source of low-frequency signals. If potential low-frequency signals are identified through M-SSA, statistical testing (section 2.3.4) then is used to quantify the likelihood that the signal (not its oscillation period) is, in fact, random. We then document that signal’s behavior (sections 2.3.6 and 2.3.7) if its statistical-significance results are robust.

#### 2.3.4 Assessing statistical significance of the identified signal

To assess the unlikelihood that the low-frequency modes identified, and properties associated with them, are not a random pattern of a noisy data set, a red-noise model (1) is fitted to the non-filtered time series of each index.1

where *x*^*n*^ is the simulated value of a given index at time *n*; *x*^*n*+1^ is its value at time *n+*1; *w* is a random number drawn from a normal distribution with zero mean and unit variance; parameters *a* and *σ* are computed from such fits by linear regression. The red-noise model generates surrogate time series. An extended matrix of these surrogate indices then is generated, to which M-SSA is applied - steps consistent with treatment of the original time series.

By construction, cross-correlations between any time series of this collection of randomly generated surrogate values would be zero at any lag. Finding any patterns, or modes, of variability in the extended surrogate matrix would be a product of chance. It is this chance that we want to evaluate in order to compare with patterns found in the extended matrix of real data.

The range of mean variances of modes identified in the surrogate data sets is determined. The range of standard uncertainty, the 95% confidence level, sets an envelope of uncertainty by which comparison can be made with results of M-SSA applied to the real data. Patterns of shared variability found among time series in the network of “real data” are considered to be statistically significant only if these “real-data” modes lie outside the envelope of values predicted by multiple surrogate simulations of this data set generated by model (1). If they do fall outside this envelope, as defined by the surrogate data, there is a presumed 5% chance or less that the occurrence of this signal, as represented by the leading modes of variability, is due to mere random chance. In the original stadium-wave study for 20^th^ century observational data, this chance of the identified signal being due to random chance was computed to be less than 3%.

#### 2.3.5 Estimating error bars

We further test for the chance that our signal may be a product only of random chance by estimating error bars for the modes. Attached to the mean variances (of identified modes) plotted on the M-SSA spectrum, error bars will serve to further test results from real data against those results from surrogate data. Here we estimate the spread of the eigenvalues, or error-bar length, using the 95% spread of variances obtained by the red-noise model. Each error bar attached to the mean variance on the M-SSA plot (see Figure 1a) can be considered to be the observed variance of the real-data’s mode plus/minus the standard deviation of the variances generated by the red-noise data (section 2.3.4).

To calculate the error bars, the variance of the mode is multiplied by the square root of 2/N*, where N* = the number of degrees of freedom. N* is estimated from the formula of (Bretherton et al. 1999): N* = *N*(1 − *r*^2^)/(1 + *r*^2^), where *N* is the length of each time series in the index set. In the earlier stadium-wave studies, where annually (boreal-winter) sampled, 100-year time series for each index were auto-correlated. The autocorrelation plots showed that the maximum autocorrelation after one year among the eight indices was *r*≈0.65. Using this auto-correlation value of *r*≈0.65 in the Bretherton formula^e^, the effective number of degrees of freedom was estimated to be 40. From this, the projected decorrelation time is N/N* = 100/40 = 2.5. This is considered the amount of time after which each data point can be considered independent from the ones preceding and following it. For the longer time series used in the control runs, where N was not equal to 100, the formula (2/N*)^1/2^ was adjusted accordingly.

#### 2.3.6 Visualizing the patterns of variability – reconstructed components (RCs)

M-SSA modes are represented in their original index space by reconstructed components (RCs). More specifically, a RC is effectively the narrow-band filtered version of an original index time series. With its construction, variability related to a given mode within a given index is easily visualized. The sum of RCs of each M-SSA mode of an index is identical to the index’s original time series. We generate RCs of the individual indices for each of the leading modes and plot them (Figure 1c). Time scale of variability is considered for the leading modes. In the case of the original stadium-wave, RCs for the leading two modes share similar time scales of variability. They were combined and their result normalized. This was that signal’s filter.

#### 2.3.7 Computing the filtered signal and visualizing its propagation

We see that the original stadium-wave-signal’s filter was constructed by summing RCs for the M-SSA “pair” of modes - leading modes one and two. Important to note is that while that signal’s identified “pair” cannot be statistically justified as an oscillatory pair, it is these two leading modes that together capture the spatiotemporal complexity of the secularly varying hemispheric-wide pattern.

The normalized RC sum is used as a sieve, of sorts, to extract the stadium-wave signal from the original time series of the network indices. If different channels of this computed filtered signal have non-zero phase lags, this would suggest a propagating signature. Indeed, non-zero lags were identified between indices of the original stadium-wave network. Normalized RCs of this joint signal are plotted in Figure 1d, reflecting the signal’s propagation through the hemispherically spanning collection of 20^th^ century indices.

We make note that additional tests were applied to the index collection in the original study to further test for robustness of results and to verify that this identified signal was expressed to one degree or another in all indices of the network (see Wyatt et al. 2011). Furthermore, recognizing that correlation cannot imply causation, numerous observational and model studies that support index coupling within the network were invoked in previous stadium-wave studies, their details described in (Wyatt et al. 2011) and (Wyatt (2012); submitted manuscript (2013)). Support appears strong for the hemispheric-wide existence of this secularly varying, propagating signal. It is this signal we seek in the current study. If such cannot be found, we will consider, in addition, any statistically significant signal propagation whose variability is inter-decadal-scale or larger, perhaps finding that models generate their own versions of stadium wave behavior.

Using the described methods and standards by which to judge results, we found the following outcomes in our model-generated data.

## 3 Results

In total, 60 model runs were analyzed. Of these, 20 runs showed at least one mode of variability to be significant at the p < 5% level. Most of these leading modes were single modes. And of those single modes, no propagating signal among the indices was found. Only five model runs showed evidence of a pair. Three of these five exhibited high frequency variability – bi-annual to sub-decadal, one with non-stationary trends (IAP_fgoals_1_0_g_20csm_run1) – but none were consistent with stadium-wave propagation, observed or otherwise. Two of the five identified RC-pairs varied at a relatively a low-frequency time scale – 35-year variability. Further testing on this pair showed no signal propagation. Thus, of all runs evaluated from the CMIP data base, none reproduced a statistically significant, hemispherically propagating signal. We found no stadium wave analogous to that found in observational and proxy data. We found no stadium wave of any kind.

Table 2 shows model results for 20^th^ century analyses. Table 3 shows model results for control (or long) runs. Table 4 summarizes those models for which at least one mode of statistically significant variability could be identified.

**Table 2:** **20thc model results (total of 50 runs of 20**^**th**^**-century simulations)**

Model	Run	Sample interval	Significant RCs	Grouping character	Frequency/Propagation traits
BCCR_bcm2_0	1	Annual	None		
BCCR_bcm2_0	1	5y	None		
CCCMA_cgcm3	1	Annual	RC1	Single	No propagation
CCCMA_cgcm3	1	5y	None		
CNRM_cm3	1	Annual	RCs 1,2,3	RCs 1,2 = pair	Bi-annual
				RC3=single	
CNRM_cm3	1	5y	None		
CSIRO_mk3	1	Annual	RCs 3,4,5,6,7	RCs 6,7 pair	Bi-annual
				All others single	Non-stationary
CSIRO_mk3	1	5y	RC1	Single	No propagation
CSIRO_mk3	2	Annual	None		
CSIRO_mk3	2	5y	None		
GFDL_2_0	1	Annual	RCs 1,2	Pair	Non-stationary
					No propagation
GFDL_2_0	1	5y	RCs 1,2	Pair	No propagation
GFDL_2_0	2	Annual	RC3	Single	No propagation
					Non-stationary
GFDL_2_0	2	5y	None		
GFDL_2_1	1	Annual	None		
GFDL_2_1	1	5y	None		
GFDL_2_1	3	Annual	None		
GFDL_2_1	3	5y	Rcs 1,2	Pair	No propagation
GISS_aom	1	Annual	None		
GISS_aom	1	5y	None		
GISS_aom	2	Annual	None		
GISS_aom	2	5y	None		
GISS_e_h	1	Annual	None		
GISS_e_h	1	5y	None		
GISS_e_r	1	Annual	None		
GISS_e_r	1	5y	None		
IAP_fgoals1_0_g	1	Annual	RCs 1,2,3	Singles	No propagation
IAP_fgoals1_0_g	1	5y	RC1	Single	No propagation
INGV_echam4	1	Annual	None		
INGV_echam4	1	5y	None		
INMcm3_0	1	Annual	None		
INMcm3_0	1	5y	None		
IPSL_cm4	1	Annual	None		
IPSL_cm4	1	5y	None		
MIROC3_2_hires	1	Annual	None		
MIROC3_2_hires	1	5y	None		
MIROC3_2_medres	1	Annual	None		
MIROC3_2_medres	1	5y	None		
MIUB_echo_g	2	Annual	RCs 1,2,3 (4,5 marginal)	Singles	high-frequency
					non-stationary
MIUB_echo_g	2	5y	RCs 1,2 marginal	Singles	No propagation
MPI_echam	1	Annual	None		
MPI_echam	1	5y	None		
MRI_cgcn_2_3_2	1	Annual	None		
MRI_cgcn_2_3_2	1	5y	None		
NCAR_CCSM3_0	1	Annual	None		
NCAR_CCSM3	1	5y	None		
NCAR_pcm1	1	Annual	RC3		high-frequency
					non-stationary
NCAR_pcm1	1	5y	None		
UKMO_hadcm3	1	Annual	RCs 1,2,3 (all marginal)	Singles	No propagation
UKMO_hadcm3	1	5y	None		

All statistically significant modes were considered (p < 5% level). Of the statistically significant single modes, no low-frequency (decadal or longer) modes showed both propagation and stationarity. Five of the fifty runs showed significance (p < 5% level) for a pair of leading modes. Despite passing the red-noise significance test, none showed a propagating signal.

**Table 3:** **Control models (total of 10 runs of Pre-industrial simulations)**

Model	Run	Sampling interval	Significant modes	RC Character	Comments
BCCR_bcm2_0	1	5y	none		
CCCMA_cgcm3	1	Annual	none		
CCCMA_cgcm3	1	5y	RC1	single	non-stationary
					no propagation
CNRM_cm3	1	Annual	none		
CNRM_cm3	1	5y	RC1	single	No propagation
CSIRO_mk3	1	Annual	none		
CSIRO_mk3	1	5y	RC2	single	No propagation
GFDL_2_0	1	5y	RCs 1,2	singles	No propagation
GISS_aom	1	Annual	none		
GISS_aom	1	5y	none		

No significant pairs were identified in any of the control runs analyzed. No propagation occurred in the statistically significant single modes.

**Table 4:** **Summary of all analyses whose results included at least one statistically significant mode (p < 5%)**

RC #	Group	Period	Model	Experiment	Run	Significant w/ annual sampling	Significant w/ 5y rm sampling	Comments
1	single	~70y	**CCMA_cgcm3**	**20c**	1	yes	no	**no propagation**
1,2	pair	bi-annual	**CNRM_cm3**	**20c**	1	yes	no	high-frequency
								**no propagation**
3	single	~25y	**CNRM_cm3**	**20c**	1	yes	no	**no propagation**
								non-stationary
3	single	sub-decadal	**CSIRO_mk3**	**20c**	1	yes	no	**no propagation**
								non-stationary
5	single	sub-decadal	**CSIRO_mk3**	**20c**	1	yes	no	**no propagation**
								non-stationary
6,7	pair	bi-annual	**CSIRO_mk3**	**20c**	1	yes	no	**no propagation**
								non-stationary
1	single	~70y	**CSIRO_mk3**	**20c**	1	no	yes	**no propagation**
1,2	pair	~35y	**GFDL_2_0**	**20c**	1	marginal	yes	**no propagation**
1,2	pair	~35y	**GFDL_2_1**	**20c**	3	no	marginal	**no propagation**
1	single	~100y	**IAP_fgoals_1_0**	**20c**	1	yes	yes	**no propagation**
2,3	pair	bi-annual	**IAP_fgoals_1_0**	**20c**	1	yes	no	non-stationary
1	single	inter-annual	**MIUB_echo_0**	**20c**	2	yes		**high-frequency**
								**non-stationary**
								**no propagation**
1	single	~60y	**MIUB_echo_0**	**20c**	2		yes	**no propagation**
2	single	~60y	**MIUB_echo_0**	**20c**	2	yes	no	**no propagation**
3	single	~25y	**MIUB_echo_0**	**20c**	2	yes	no	**no propagation**
3	single	~55y	**UKMO_hadcm**	**20c**	1	marginal	no	**no propagation**
1	single	~50y	**CNRM_cm3**	**control**	1	n/a	marginal	**no propagation**
2	single	~25y	**CSIRO_mk3**	**control**	1	n/a	yes	**no propagation**
1	single	~55-75y	**GFDL_2_0**	**control**	1	n/a	yes	**no propagation**
2	single	~25y	**GFDL_2_0**	**control**	1	n/a	yes	**no propagation**

Of all analyses with statistically significant modes identified, none capture a propagating signal.

### 3.1 Results of select models

Results for select models are detailed in this section. These results are representative of results of all analyses performed. Their plots are shown in Figures 2, 3, 4, 5, 6, 7, 8, 9, 10, 11 and 12.

**Figure 2:**
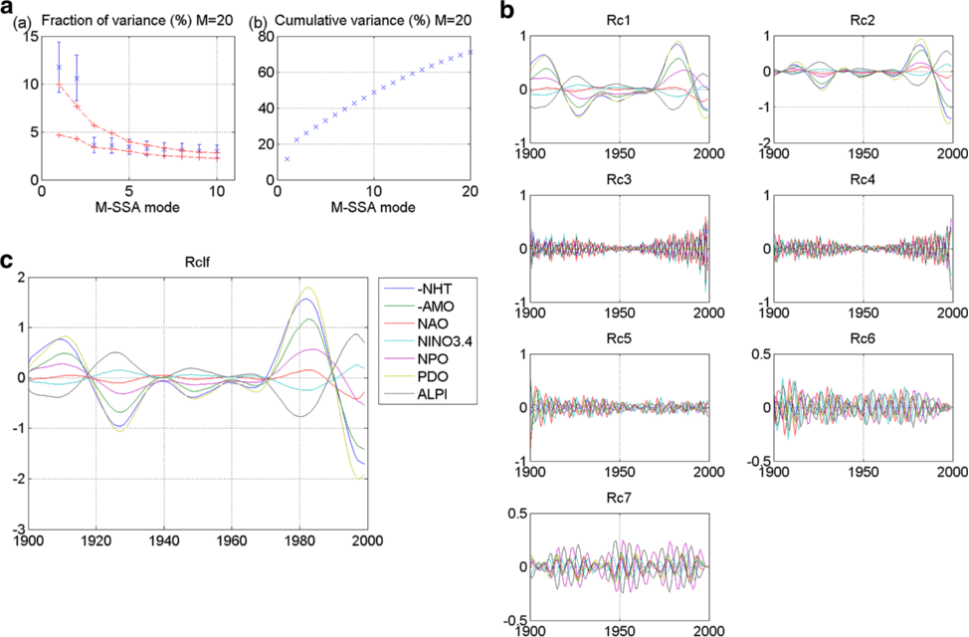
**Output from certain GFDL 20thc models showed promise.** While the two M-SSA-identified leading modes showed potential for an oscillatory pair in GFDL_2_0_20thc Run 1, (**a**) as deduced from the two leading modes (at p < 5% significance) being mostly outside the red-noise-established envelope of uncertainty in the M-SSA spectrum; (**b**) and the RCs of leading modes one and two showing similar periodicity; (**c**) their failure to produce a propagating signal is due to the normalized RCs of the combined modes being in exact phasing, not offset (see text). Thus, this cannot be considered the “stadium wave”.

**Figure 3:**
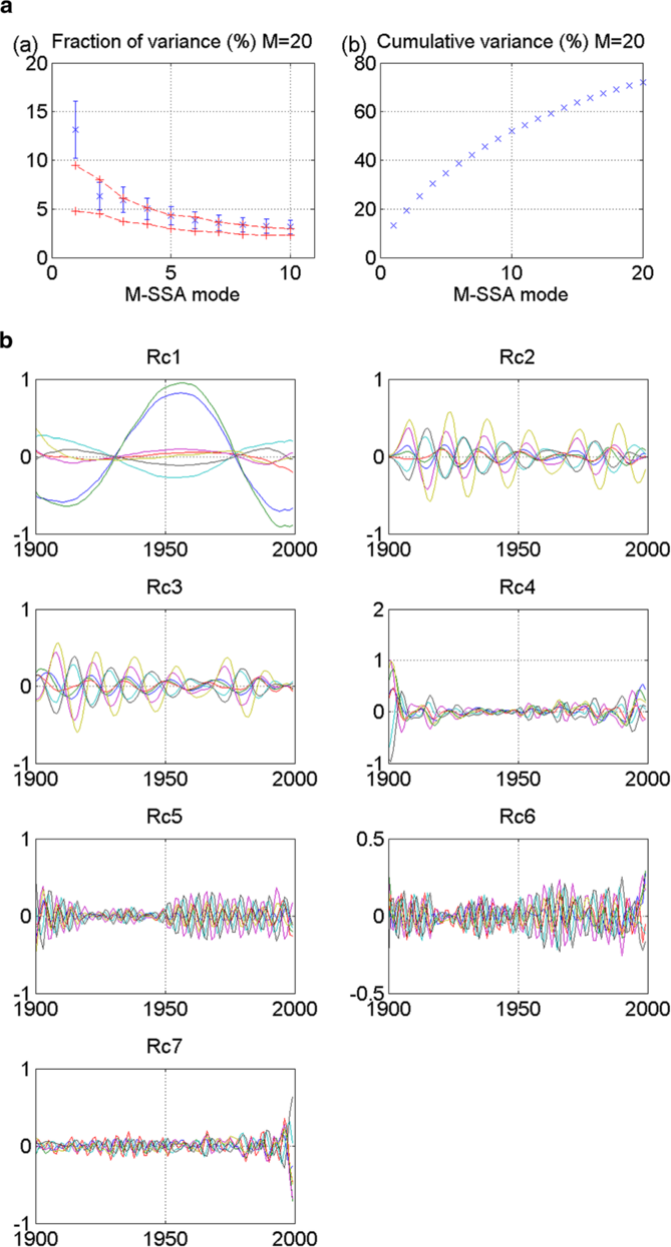
**CCCMA 20thc run1: No stadium-wave signature.** (**a**) Two plots are given. The first is the M-SSA spectrum for this model. It shows leading mode 1 is significant. There is no indication of a pair. The second plot in (**a**) is the cumulative variance of the first 20 modes identified. (**b**) Shows the RCs for each mode. Mode 1 is likely a forced radiative signal.

**Figure 4:**
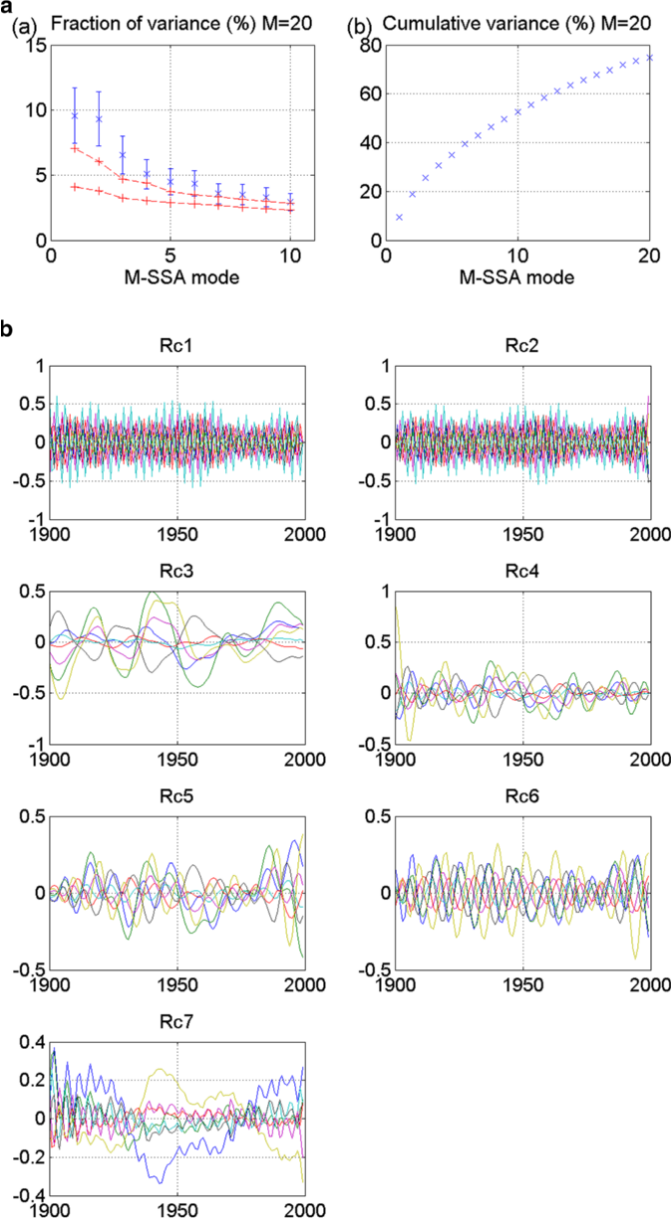
**CNRM 20thc run 1: No stadium-wave signature.** (**a**) Two plots are given. The first is the M-SSA spectrum for this model. It shows leading modes 1&2 to be outside the red-noise envelope. Their error bars overlap; yet there is no separation from the remaining modes. The second plot in (**a**) shows the cumulative variance accounted for by the first twenty modes. (**b**) Shows RCs of the individual modes. RCs for modes one and two are high-frequency modes. RCs for mode three are decadal-plus, but no propagation.

**Figure 5:**
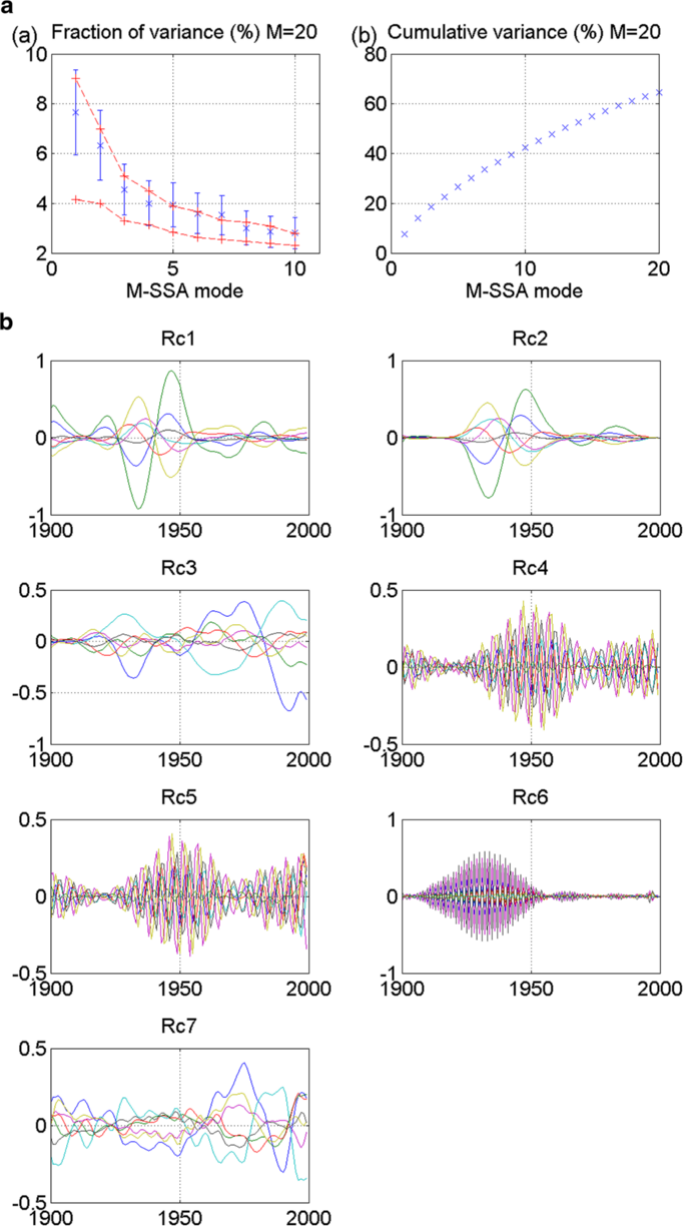
**GISS_aom 20thc run 2: No stadium-wave signature.** (**a**) Two plots are given. The first is the M-SSA spectrum for this model. No modes fall outside the red-noise envelope; thus no significant mode can be identified. The second plot in (**a**) shows the cumulative variance accounted for by the first twenty modes. (**b**) Shows the RCs. The first two are interdecadal; yet their phasing is in-phase. The signal, therefore, does not propagate through the indices. All tests for a stadium-wave signal are failed for this model’s data set.

**Figure 6:**
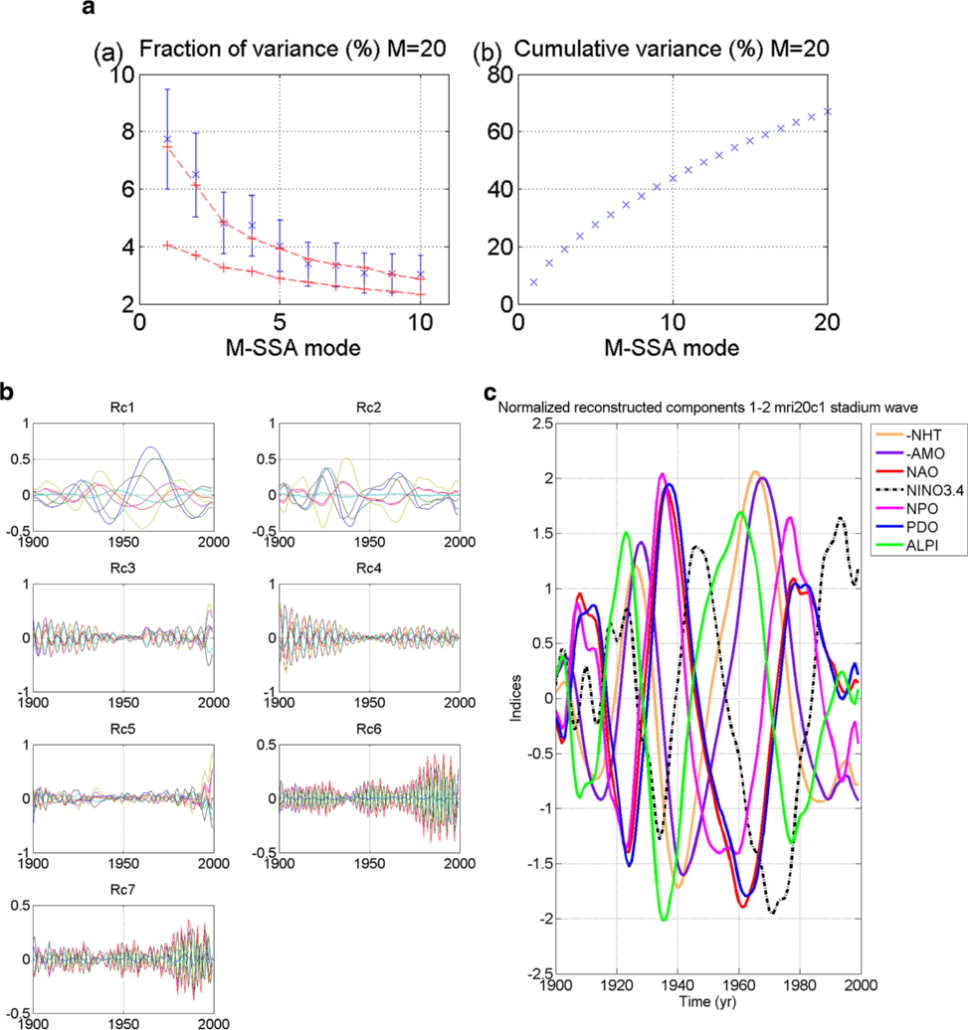
**MRI_cgcm_2_3_2 20thc run 1: No statistically significant stadium-wave signature.** (**a**) Two plots are given. The first is the M-SSA spectrum for this model. No modes fall outside the red-noise envelope and no modes are well-separated from others; thus no significant mode can be identified. The second plot in (**a**) shows the cumulative variance accounted for by the first twenty modes. (**b**) Shows the RCs of individual modes one through seven. (**c**) Shows the statistically non-significant “stadium-wave-like” propagation of normalized RCs of joint modes one and two. Many caveats are attached to this “wave”, lack of statistical significance being only one. The spatial pattern differs from the original stadium wave, with ALPI non-aligned with NPO and PDO as an example. Temporal character of NINO is unlike that of the original ‘wave’. For the entire mri_20thc ‘wave’, time scale of variability progressively increases as signal progresses through the time series. Sequence of signal propagation appears to fall apart with time.

**Figure 7:**
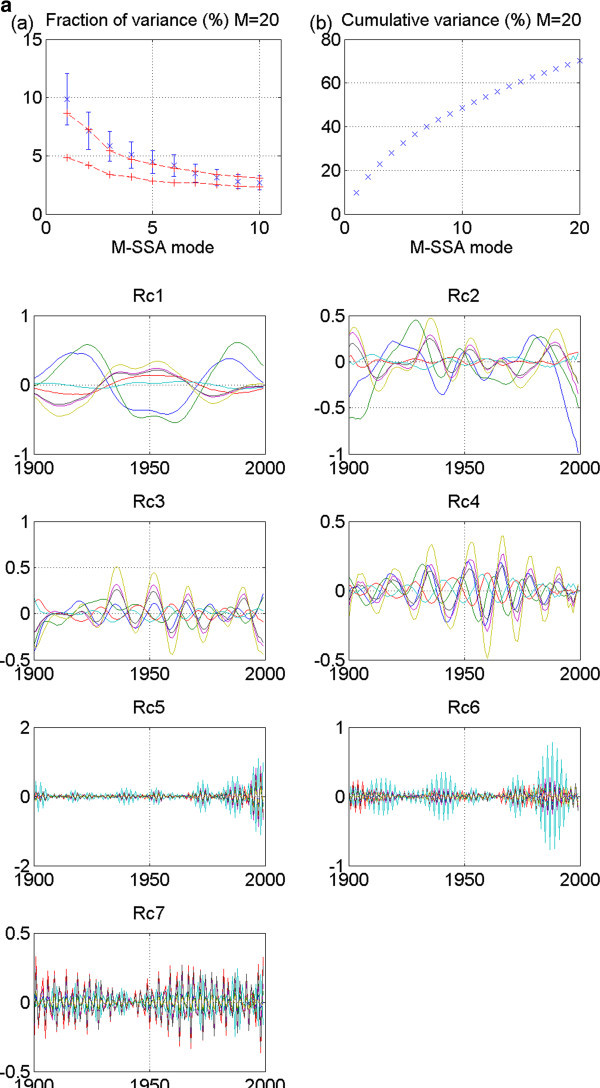
**NCAR_CCSM3 20**^**th**^**c run 1: No stadium-wave signature.** (**a**) Two plots are given. The first is the M-SSA spectrum for this model. No modes fall outside the red-noise envelope and no modes are well-separated from others; thus no significant mode can be identified. The second plot in (**a**) shows the cumulative variance accounted for by the first twenty modes. (**b**) Shows the RCs for this model. RC1 has a multidecadal character, but it is not a propagating signal. It is a moot point, as none of the modes fall outside the red-noise envelope.

**Figure 8:**
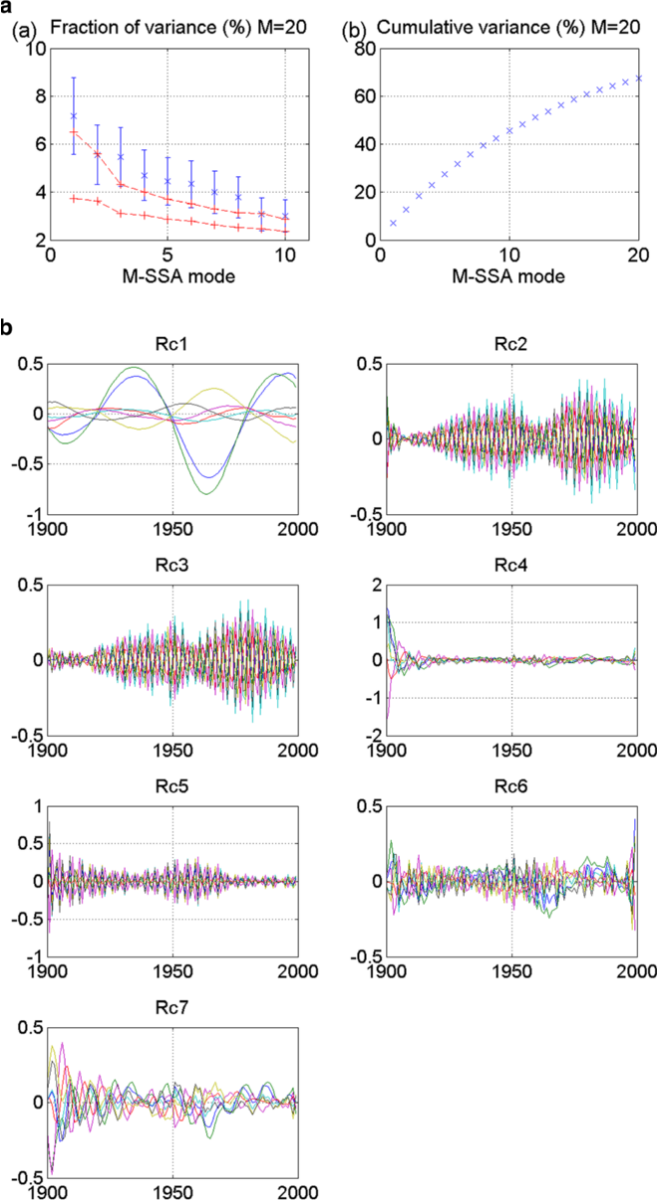
**NCAR pcm1 20thc run 1: No stadium-wave signature.** (**a**) Two plots are given. The first is the M-SSA spectrum for this model. No modes fall outside the red-noise envelope and no modes are well-separated from others; thus no significant mode can be identified. The second plot in (a) shows the cumulative variance accounted for by the first twenty modes. (**b**) Shows the RCs for this model. As in the other NCAR model (Figure 7), RC1 exhibits a multidecadal tempo. It is a single mode with no propagating signal. It is a moot point, as none of the modes fall outside the red-noise envelope.

**Figure 9:**
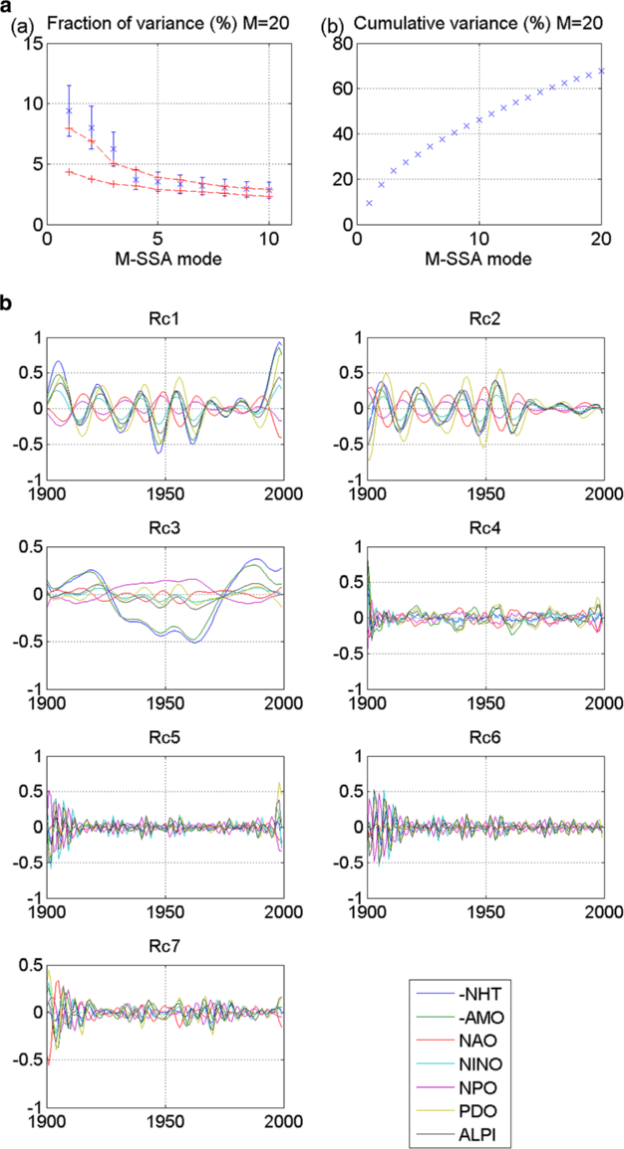
**UKMO_hadcm3 20thc run 1: No stadium-wave signature.** (**a**) Two plots are given. The first is the M-SSA spectrum for this model. The first three modes in UKMO_hadcm3 20thc run 1 overlap and are fairly well separated from the remaining modes; yet error bars for these three are not completely out of the envelope of uncertainty. The second plot in (**a**) shows the cumulative variance accounted for by the first twenty modes. (**b**) Shows the RCs for this model. Even if we were to consider the first few modes as being significant, one can see that modes one and two are higher frequency modes (not what we are looking for) and those modes display some non-stationarity in the data (again, not what we are looking for). Mode 3 exhibits multidecadal behavior; yet no propagation is evident.

**Figure 10:**
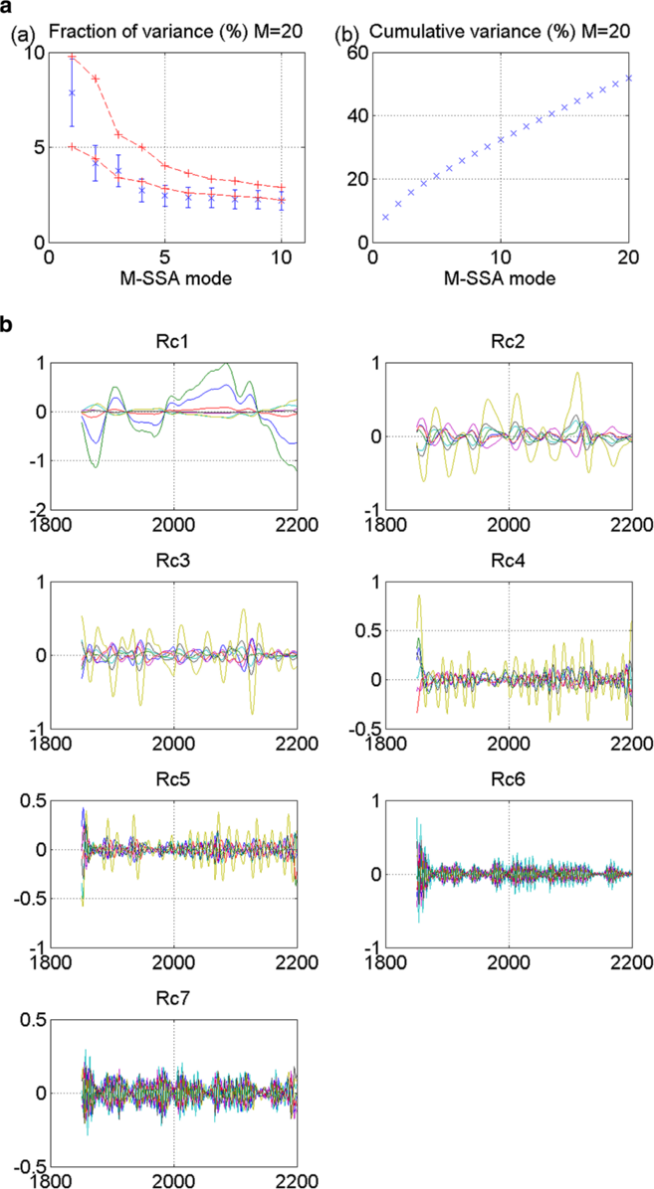
**CCCMA_cgcm3_control run1: No stadium-wave signature.** (**a**) Two plots are given. The first is the M-SSA spectrum for this model. No modes fall outside the red-noise envelope. The second plot in (**a**) shows the cumulative variance accounted for by the first twenty modes. (**b**) Shows the RCs for this model. Most modes display high frequency variability, with the first mode showing some of the indices displaying a multidecadal timescale; while the amplitude of the other index RCs is minimal to zero. There is no signal propagation.

**Figure 11:**
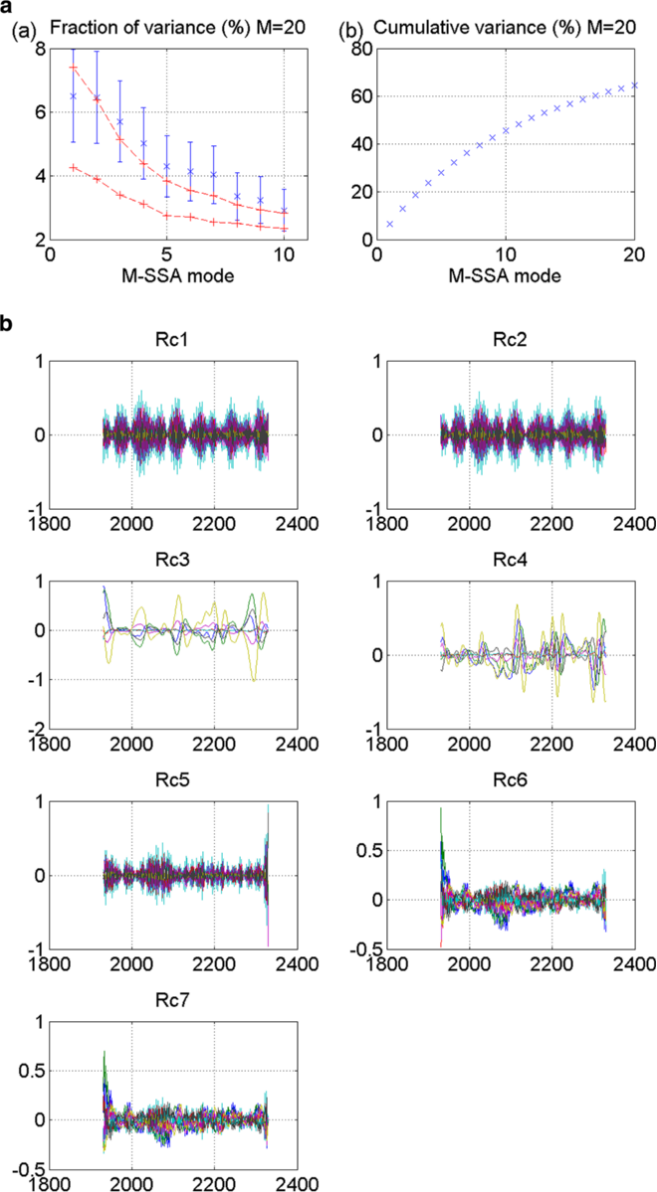
**CNRM_cm3_control run1: No stadium-wave signature.** (**a**) Two plots are given. The first is the M-SSA spectrum for this model. No modes fall outside the red-noise envelope. Nor are any modes widely separated from the others. The second plot in (**a**) shows the cumulative variance accounted for by the first twenty modes. (**b**) Shows the RCs for this model. Most modes display high frequency variability. No semblance of a stadium-wave signal is seen.

**Figure 12:**
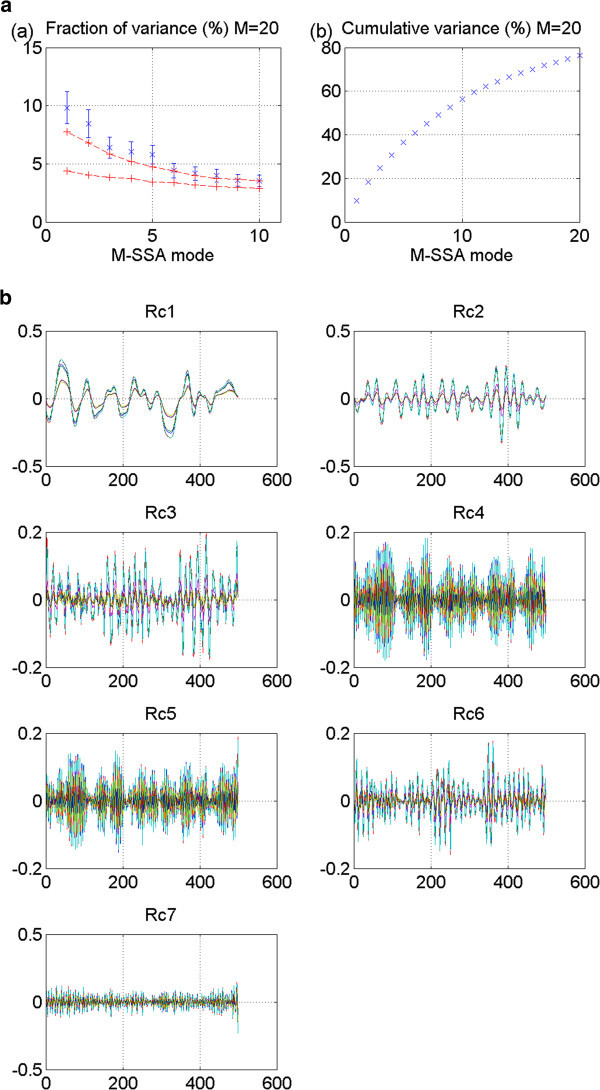
**GFDL_2_1 control run1 (smoothed with 5-y running mean): No stadium-wave signature.** (**a**) Two plots are given. The first is the M-SSA spectrum for this model. Modes 1&2 fall outside the red-noise envelope. Their error bars overlap and they are somewhat separated from the remaining modes. The second plot in (**a**) shows the cumulative variance accounted for by the first twenty modes. (**b**) Shows the RCs for this model. Most modes display high frequency variability. Mode one reflects a multidecadal character, but mode two is of a different, much higher frequency. No oscillatory or propagating signal can be identified.

Model results from run 1 of the 20^th^-century experiment of GFDL_2_0 appeared promising at first glance. The M-SSA spectrum (Figure 2a) indicated two leading modes mostly outside the red-noise envelope that were well-separated from the remaining modes. Their error bars overlapped, indicating similar mean variances. We show plots of reconstructed components (RCs) of the indices for each of seven modes of variability. Those for modes one and two are the ones of interest. They appear to display similar periodicities (Figure 2b). We combined the RCs for modes one and two to obtain the dominant signal characterizing this data set. It can be seen that signal expression in each of the seven indices occurs either in-phase or 180° out-of-phase. There is no signal propagation through the indices (Figure 2c).

The M-SSA plot for the 20^th^-century experiment for model CCCMA_cgcm3 run 1 is shown in Figure 3a. Mode one falls outside the red-noise envelope, indicating its statistical significance. There is no second mode of a similar mean value. This single mode reflected in the companion RC plot (Figure 3b) shows a multidecadal character. It is likely a radiative signal. All other indices show only a slight undulation, and all indices are either in-phase or 180° out-of-phase. No stadium-wave signal propagation is indicated.

For the CNRM 20^th^-century model, run 1, three modes fall outside the red-noise envelope of the M-SSA spectrum and several others are almost outside it (Figure 4a). The first two overlap. In fact, they are parallel. Their mean values are essentially identical. A glance at their associated RCs reveals they are high-frequency modes, likely seasonal. These are not candidates for the signal we seek. Other modes might be considered; yet no pair meets the criterion of being widely separated from their neighbors. Furthermore, the associated RCs show no signals of similar periodicities. Nor is there any single mode that is widely separated from the others. Thus, again, we detect no stadium-wave signal.

GISS_aom is the next 20^th^ century model whose results are shown. These results are for run 2. No modes fall outside the red-noise envelope, rendering the results negative (Figure 5a). Non-stationarity of data characterizes all associated RCs (Figure 5b). There is no stadium-wave signal found in this data set.

Twentieth-century runs of models MRI_cgcm_2_3_2 (run 1), NCAR_CCSM3_0 (run 3), and NCAR_pcm1 (run 1) show no significant modes in their results (Figures 6, 7 and 8). No mode falls outside the red-noise envelope. Thus, no stadium-wave signal is identified, despite the fact that the first modes of each of the models show a multidecadal character. For the two versions of the NCAR models (Figures 7 and 8), the first mode is a single one. A strong expression exists in the NHT and AMO, but all indices are either in-phase or 180° out-of-phase. No propagation is evident.

In the MRI model, no modes can be considered statistically significant. Nor are the leading modes one and two well separated from the remaining modes. This is enough to disqualify them from our search; yet the similar multidecadal tempos of modes one and two are interesting. Normalized RCs of the sum of these two modes (Figure 6c) suggest signal propagation; although not analogous to the original ‘wave’. That aside, it is statistically non-significant. And additional caveats prevent its candidacy. It displays weak spatial and temporal coherence. This example, while intriguing, cannot be considered a stadium-wave signal.

The UKMO_hadcm3 20^th^-century model results (run 1) show three leading modes whose error bars overlap, are distinct from the remaining modes, and are almost fully outside the red-noise envelope (Figure 9a). Despite these positive results, the first two RCs show decadal variability with non-stationarity in the time series. The third RC is a multidecadal mode; yet it is a single one with no propagation (Figure 9b). Thus, no stadium wave is found in this data pool.

Results from control experiments CCCMA_cgcm3 (run 1) and CNRM_cm3 (run 1) are shown in Figures 10 and 11. No modes fall outside the red-noise envelope. Centennial-scale variability characterizes mode one of the CCCMA model and a more inter-decadal character can be seen in modes 2 and 3. The remaining modes in this model and all the modes of the CNRM model are high-frequency modes. No results for these models suggest a stadium-wave signal.

The last model discussed here is the control experiment of GFDL_2_1 (Figure 12). The leading two modes of variability fall outside the red-noise envelope, overlap, and are mostly separated from the remaining modes (Figure 12a). RCs for mode one indicate a multidecadal tempo; RCs for mode two reflect a higher frequency (Figure 12b). No propagation of signal is evident in any mode. Again, no stadium-wave signal is detected.

## 4 Summary and discussion

We analyzed networks of reconstructed indices from model-generated data from the third Coupled Model Intercomparison Project (CMIP3). Our goal in so doing was to determine if a hemispherically propagating climate signal, previously detected at secularly varying time scales in analogous index networks reconstructed from 20^th^-century instrumental data, could be identified. That secular-scale signal found in 20^th^ century instrumental data, termed the ‘stadium wave’, was characterized by two leading modes of low-frequency variability, whose normalized sum of RCs documented a spatiotemporally complex signature propagating across the Northern Hemisphere. Subsequent to identification of stadium-wave behavior in 20^th^-century instrumental data (Wyatt et al. 2011), analogous stadium-wave behavior was found in a companion study, which was based on a variety of spatially and dynamically diverse instrumental-data sets, and temporally expanded proxy-data sets (Wyatt (2012); submitted manuscript (2013)).

In the present model-data based study, we adapted methods identical to those used in two prior companion studies. And in this study, we were unable to identify a ‘wave’ analogous to the original stadium wave detected in observations. While results of some model experiments hinted at signal propagation, none of these signals were found to be statistically significant or stationary. Furthermore, none were spatially or temporally analogous to the “wave” found in observational and proxy data.

As a whole, co-variability among indices of seven-member networks assembled from data generated by models of the CMIP3-ensemble simulations appears to be dominated by high-frequency fluctuations, with the most dominant ones at biannual to sub-decadal periodicities. These are often marked by non-stationarity across the time interval evaluated. Radiative-forcing signatures (in the NHT, in particular) are apparent at the secular-scale in some runs. These signatures are characterized by a long, non-periodic variation with at most only one full cycle in a one-hundred-year span. Statistically significant signal propagation is absent among the model sets analyzed. These features stand in contrast to those of the observed stadium wave. Furthermore, consistency of results from model to model, or even model-run to model-run of the same model was not an outstanding feature in our analysis; while in previous stadium-wave studies, the same secularly varying, hemispherically propagating, spatio-temporal signal emerged consistently, time-after-time, in one index set after another.

Negative results from a study such as this are not capable of definitively claiming the existence of deficiencies in model design. We conducted this experiment not to evaluate model design, nor to support or refute the stadium-wave signal detected in other data sets in previous studies. This research was conducted simply to answer the question, ‘is the hemispherically propagating climate signal found in observational and proxy data sets also found in model-simulated data sets of the CMIP3 ensemble?’ The answer is ‘no’. With this unexpected result, we are left to question, why not. We submit this question invites curiosity.

Our methods used in this study to generate data sets may be the culprit. Were our index reconstructions sound? Or did the omission of the index, AT (atmospheric-mass transfer anomalies), from our reconstructed networks negatively affect the modeled outcome? Perhaps these possible deficiencies deserve closer scrutiny. As far as the former, we did test each reconstruction code designed for the model data. We did so by substituting “real” data for the modeled data. Our results were good. But this does not rule out the possibility of undetected small errors that grew when combined with other small index errors. As far as the latter caveat, as discussed in section 2.2.2, the ‘stadium-wave’ signal emerged as statistically significant in data sets with the AT index omitted; yet that statistical significance was not as robust as with AT’s inclusion. This may have made a difference when using the model-simulated indices. Perhaps we performed too few analyses. Or perhaps our studies suffer from deficiencies we have yet to recognize.

But we also posit that these results - the failure of the model-generated data to yield a propagating signal – might speak to a missing ingredient in modeling design. We offer that this missing ingredient includes dynamics that play significant roles in hemispheric signal propagation, in linking one regional circulation pattern with another.

For example, in observation-based studies, geographical positioning of large-scale oceanic and atmospheric centers-of-action (COA)^f^ has been found to be critical to connectivity among regional circulations, establishing communication links that help make sense of intra-hemispheric signal propagation (Kirov and Georgieva 2002; Polonsky et al. 2004; Dima and Lohmann 2007, Wang et al. 2007; Msadek et al. 2010 for examples). For instance, the Icelandic and Aleutian Lows shift longitudinally and latitudinally (Dima and Lohmann 2007; Georgieva et al. 2007) on decadal-plus timescales, influencing, among other things, dominant basin-scale wind flow. Inter-basin connectivity modifies accordingly.

One related example is drawn from research by (Wang et al. 2007). They find that COA migrations generate intervals when climate patterns over the North Pacific and over the Eurasian continent upstream are linked. Likewise, regions downstream are linked. These linkages can be traced to an enhanced Pacific North American (PNA) pattern and to an eastwardly extended jet stream. These nuanced factors influence El Nino’s relationship with the Aleutian Low Pressure system.

Another example can be found in works by (Sugimoto and Hanawa 2009) and (Frankignoul et al. 2011). These works suggest that low-frequency latitudinal shifts in atmospheric COAs, influence migrations of western-boundary currents and their extensions (ocean-gyre frontal boundaries), with consequent impact on western-boundary dynamics and air-sea interaction (Kwon et al. 2010; Frankignoul et al. 2011). Ocean-atmospheric dynamics related to western-boundary currents are not typically well modeled, spatial resolution and representation of heat flux out of the ocean being among the limiting factors (Dong and Kelly 2004; Kelly and Dong 2004; Kelly et al. 2010). We offer this limitation may play a role in the contrasting results of the stadium-wave studies, observation-based versus model-based.

Geographical placement of the Arctic High (Kwok 2011) presents another illustration of this theme. Mean and shifting geographical placements of the polar high-pressure system play strong roles in Arctic sea-ice dynamics, freshwater balance, and by extension, in climate response Wyatt (2012); submitted manuscript (2013). Most models inadequately simulate the Northern Hemisphere’s polar-high placement. (Kwok 2011) evaluated simulations of Arctic sea-ice motion in the CMIP3 collection. Sea-ice motion is largely wind-driven. The impacts of these winds are a function of, among other things, the geographical placement of the polar-high. This placement indirectly scripts regional sea-ice inventory, distribution, spatial pattern of ice thickness, and sea-ice export. Kwok examined groups of models. He found the overall CMIP3 simulations of sea-ice dynamics and related features to be poor, with some models performing better than others. He suggests the culprit is the significant displacement of the mean high-pressure pattern in the southern Beaufort region. Its modeled position tends to be skewed toward the central Arctic Basin rather than its observed mean position in the southern Beaufort Sea. Misplacement of related large-scale mean features of the circulation pattern follows. Other influences on the Arctic freshwater balance derive from sea-ice extent north of the Bering Strait. This, too, has been linked to non-static geographical placement of the Aleutian Low (Niebauer 1998). And according to (Jun et al. 2008), errors related to sea-ice north of the Bering Strait are common among models: GFDL, GISS, NCAR, and UKMO.

Geographical placement of centers-of-action and dynamics of western-boundary currents are but two identified features that appear to determine whether a regional circulation pattern’s reach remains regional or extends beyond, via direct or indirect means. These features are examples of small variations begetting disproportionately large results. The classic work on network theory by sociologist Mark Granovetter (1973) points to such small links yielding profound consequences. In his seminal work, he describes the crucial role of weak ties in enlarging and stabilizing a network. He describes the phenomenon in terms of societal behavior; yet this concept applies to any network. We suggest the “weak-tie” details of inter-connectivity within the climate network may be a necessary ingredient for hemispheric signal propagation. It may be that regional patterns are well modeled. But is their connectivity equally well modeled, be that through geographical positioning of oceanic and atmospheric centers-of-action; western-boundary currents, their extensions, and their relationship to overlying jet-stream tracks; or other such “small-scale” features?

And finally, along that same vein, (Van den Berge et al. 2011) have considered connectivity among nodes when modeling climate. They invoke the influential work done by (Pecora and Carroll 1990) on non-linear systems, applying principles of synchronized theory to modeling climate. In essence, they have found that with a limited amount of information exchanged, a system’s behavior can be reconstructed. This information exchange is accomplished by connecting each variable of a model to each variable of two other models. By linking chaotic systems, synchronization of the network of systems follows (Pecora and Carroll 1990). Here, consistent with what we see in stadium-wave dynamics, links between nodes are critical to capturing the full spatio-temporal signature of the climate network.

## 5 Conclusion

Analyses performed on indices reconstructed from data generated by models archived in the CMIP3 database failed to detect a statistically significant stadium-wave climate signal. Results were the same for both 20^th^-century experiments and long-control runs of pre-industrial experiments. We cannot offer an explanation for this, only speculation.

In previous stadium-wave studies, this signal was identified for the 20^th^ century in a wide variety of geographically and dynamically diverse instrumental and proxy-reconstructed geophysical indices. Ocean-ice-atmospheric coupling is hypothesized as lying at the heart of signal propagation (Wyatt 2012; submitted manuscript (2013).

Weak ties in network behavior are critical to network stability and function. Weak ties within the climate network appear to include, among other things, geographical positioning of oceanic and atmospheric centers-of-action. These features have been shown to be poorly represented in many models. This may offer insight to a physical mechanism that may be relevant to signal propagation that appears to be missing from this suite of models.

## Endnotes

^a^Synchronization refers to the matching of rhythms of self-sustained quasi-oscillators (our intrinsically variable climate indices); whereas synchronous is distinct from synchronization. Synchronous means “same timing”. The stadium-wave signal involves a network of synchronized climate indices.

^b^Occurring one or fewer times per century.

^c^(NHT, AMO, NAO, NINO3.4, NPO, PDO, and ALPI)

^d^In the original study, we used M=20, but in addition, M was varied. No changes in outcome resulted; thus we justify use of only M=20 for the window size in this study.

^e^The majority of other indices in this network had much lower auto-correlation values, suggesting our estimated degrees-of-freedom may be on the low side, implying the statistical-significance of the stadium-wave signal in observed data may be larger than is stated here.

^f^The term ‘centers-of-action’ (COA) refers to circulation centers. In the atmosphere, the Aleutian Low and Icelandic Low are examples of COAs. In the ocean, the subpolar and subtropical gyres are COAs.
